# Genistein Inhibits Osteoclastic Differentiation of RAW 264.7 Cells via Regulation of ROS Production and Scavenging

**DOI:** 10.3390/ijms150610605

**Published:** 2014-06-12

**Authors:** Sang-Hyun Lee, Jin-Kyoung Kim, Hae-Dong Jang

**Affiliations:** Department of Food and Nutrition, Hannam University, Daejeon 305-811, Korea; E-Mails: blackbean10@naver.com (S.-H.L.); kikkne@naver.com (J.-K.K.)

**Keywords:** genistein, osteoclastic differentiation, reactive oxygen species, Nox1, mitochondrial electron transport chain system, phase II antioxidant enzymes

## Abstract

Genistein, a phytoestrogen, has been demonstrated to have a bone-sparing and antiresorptive effect. Genistein can inhibit the osteoclast formation of receptor activator of nuclear factor-κB ligand (RANKL)-induced RAW 264.7 cells by preventing the translocation of nuclear factor-κB (NF-κB), a redox-sensitive factor, to the nucleus. Therefore, the suppressive effect of genistein on the reactive oxygen species (ROS) level during osteoclast differentiation and the mechanism associated with the control of ROS levels by genistein were investigated. The cellular antioxidant capacity and inhibitory effect of genistein were confirmed. The translation and activation of nicotinamide adenine dinucleotide phosphate (NADPH) oxidase 1 (Nox1), as well as the disruption of the mitochondrial electron transport chain system were obviously suppressed by genistein in a dose-dependent manner. The induction of phase II antioxidant enzymes, such as superoxide dismutase 1 (SOD1) and heme oxygenase-1 (HO-1), was enhanced by genistein. In addition, the translational induction of nuclear factor erythroid 2-related factor 2 (Nrf2) was notably increased by genistein. These results provide that the inhibitory effects of genistein on RANKL-stimulated osteoclast differentiation is likely to be attributed to the control of ROS generation through suppressing the translation and activation of Nox1 and the disruption of the mitochondrial electron transport chain system, as well as ROS scavenging through the Nrf2-mediated induction of phase II antioxidant enzymes, such as SOD1 and HO-1.

## 1. Introduction

The bone remodeling process consists of the resorption of mineralized bone by osteoclasts and bone formation by osteoblasts [1]. Bone resorption is related to osteoclast formation and tartrate-resistant acid phosphatase (TRAP) activity, whereas bone formation is associated with osteoblastic proliferation, alkaline phosphatase activity, collagen synthesis and mineralization [2]. Thus, excessive osteoclastic bone resorption relative to osteoblastic bone formation often results in osteopenic diseases, including rheumatoid arthritis and osteoporosis [3]. Osteoclasts are bone-resorbing multinucleated cells formed by the fusion of their mononuclear precursors as monocytes and macrophages [4]. Osteoclast differentiation of precursors can be stimulated by the receptor activator of nuclear factor-κB ligand (RANKL) produced by osteoblasts [5]. It is well known that the binding of RANKL to its receptor induces small nontoxic amounts of reactive oxygen species (ROS) as various growth factors, as well as cytokines, including tumor necrosis factor-α [6]. A low-level increase of ROS may be required as a secondary messenger in RANKL-induced signaling pathways for osteoclast differentiation [6]. During osteoclast differentiation, ROS is likely to be endogenously produced either by nicotinamide adenine dinucleotide phosphate (NADPH) oxidase (Nox) or as byproducts of the mitochondrial electron transport chain, and the generated ROS is also removed by phase II antioxidant enzymes, such as superoxide dismutase (SOD), catalase and HO-1 [7,8]. Therefore, the control of the cellular reduction status by antioxidants may suppress osteoclast differentiation from osteoclastic precursors.

Genistein is the aglycone of genistin, which is one of the major isoflavones found in unprocessed soybeans, and it is more abundant in various soy-based products that are currently consumed around the world. Genistein has been shown to have several beneficial functions, including antioxidant [9,10], anti-inflammatory [11], immuno-modulation [12] and anti-carcinogenic effects [13,14]. Furthermore, because genistein has been demonstrated to act as a bone-sparing and antiresorptive agent, it has been considered to be a phytoestrogen [15]. This preventive action on bone loss has been reported in various animal and epidemiological studies [16,17]. According to previous *in vitro* studies, genistein stimulates osteoblastic differentiation and mineralization and inhibits osteoclast formation from pre-osteoclast cells and the bone resorption activity of osteoclasts [18,19]. During osteoclast differentiation from pre-osteoclast RAW 264.7 cells activated by RANKL, genistein can inhibit RANKL-induced inhibitor-κB (I-κB) degradation and nuclear factor-κB (NF-κB) translocation to the nucleus, resulting in the prevention of osteoclast formation [20]. This inhibitory effect of genistein on osteoclast differentiation may be associated with its potent antioxidant activity, because the NF-κB/I-κB signaling pathway is known to be redox sensitive [21]. However, little information about how the ROS level is controlled by genistein during osteoclast differentiation from osteoclastic precursors is available.

Therefore, in this study, the suppressive effect of genistein on the ROS level during osteoclast differentiation of RANKL-induced RAW 264.7 cells was investigated after confirming its cellular antioxidant capacity in HepG2 cells, and it was determined how the ROS level can be controlled in view of its production and scavenging by genistein.

## 2. Results and Discussion

### 2.1. Cellular Antioxidant Capacity of Genistein

**Figure 1:**
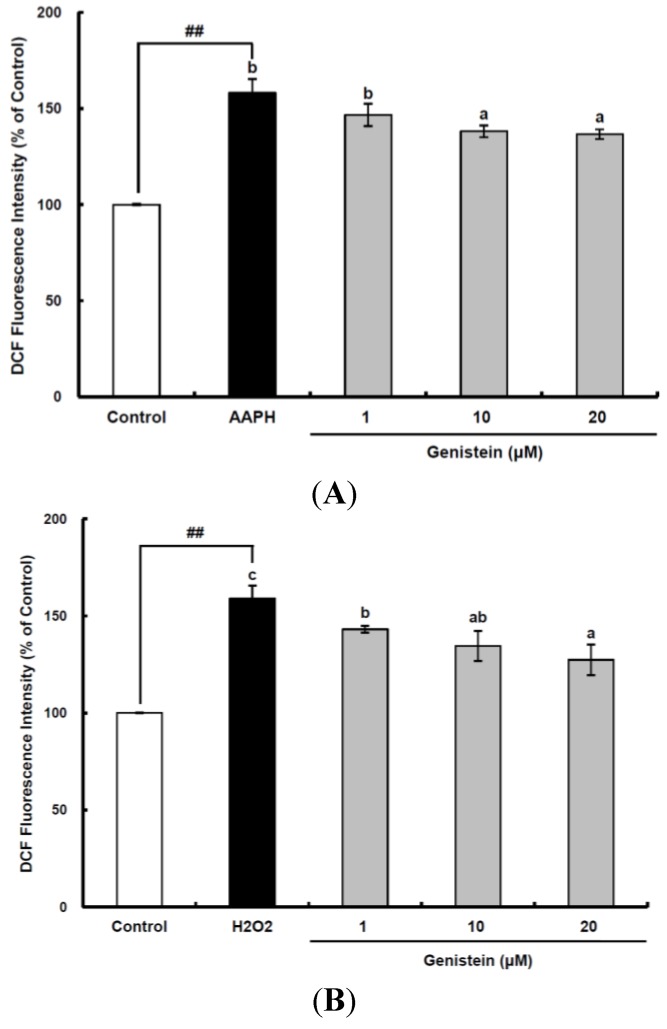
Cellular antioxidant capacity of genistein against oxidative stress induced by 2,2'-azobis(2-amidinopropane) dihydrochloride (AAPH) (**A**) and H_2_O_2_ (**B**) in HepG2 cells. HepG2 cells were first cultured in 96-well plates (5 × 10^5^/mL) with Dulbecco’s modified Eagle’s medium (DMEM) for 24 h. After the cells were incubated with different concentrations of sample dissolved in dimethylsulfoxide (DMSO) for 30 min, Hank’s balanced salt solution (HBSS), which is fluorescently stable, was then added to each well. After the cells were treated with 60 μM AAPH or 1 mM H_2_O_2_ for 30 min, 2',7'-dichlorodihydrofluorescein-diacetate (DCFH-DA) was added to the culture plates at a final concentration of 40 µM, and the cells were incubated for 30 min at 37 °C in the dark. Then, the cells were washed with HBSS and 2',7'-dichlorofluorescein (DCF) fluorescence intensity was measured at an excitation wavelength of 485 nm and an emission wavelength of 535 nm using a Tecan GENios fluorometric plate reader. Data are expressed as percentages of the value of untreated cells (mean ± standard deviation, *n* = 3). Different corresponding letters indicate significant differences at *p* *<* 0.05 by Duncan’s test. ## *p* < 0.01 *vs.* control.

The intracellular antioxidant capacities of genistein were investigated using a cellular antioxidant capacity assay. Cellular experiments were performed using the HepG2 cell line, which is an excellent biochemical and nutritional model in which many antioxidants can be assayed with less variations [22]. HepG2 cells were pre-incubated with 1–20 µM genistein for 30 min and exposed to 60 µM 2,2'-azobis(2-amidinopropane) dihydrochloride (AAPH) or 1 mM H_2_O_2_ for 30 min. The cells were then treated with 2',7'-dichlorodihydrofluorescein-diacetate (DCFH-DA), which is a fluorescent probe that detects ROS, for 30 min to measure the intracellular oxidative stress induced by AAPH or H_2_O_2_. The intracellular oxidative stress in HepG2 cells increased 158.2% and 159.1% following treatment with AAPH and H_2_O_2_, respectively, as compared to the control group (Figure 1A,B). The intracellular oxidative stress induced by AAPH in HepG2 cells was dose-dependently alleviated by genistein at 1–10 µM, whereas the intracellular oxidative stress induced by H_2_O_2_ was reduced by genistein in a dose-dependent manner at 1–20 µM. These data are consistent with another study that proved the suppressive effect of genistein on cumene hydroperoxide-induced radical generation in rat astroglioma cells [23]. When reactive oxygen radicals were produced by 500 µM cumene hydroperoxide, genistein at 1–50 µM attenuated the oxidative stress determined by DCFH-DA in a dose-dependent manner.

### 2.2. Inhibitory Effect of Genistein on the Osteoclastic Differentiation of RAW 264.7 Cells

**Figure 2:**
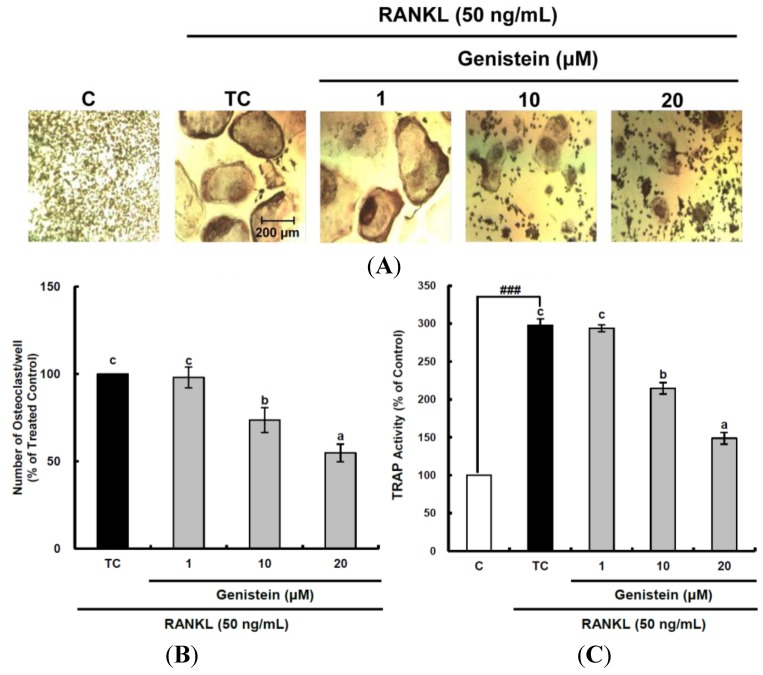
Inhibitory effects of genistein on differentiation (**A**,**B**) and tartrate-resistant acid phosphatase (TRAP) activity (**C**) of osteoclastic RAW 264.7 cells. RAW 264.7 cells were exposed to the receptor activator of nuclear factor kappa-B ligand (RANKL) (50 ng/mL) for five days in the absence and the presence of genistein. After five days in culture, the cells were fixed and stained using a leukocyte acid phosphatase kit. TRAP-positive multinucleated osteoclasts were visualized under light microphotography (**A**); TRAP-positive multi-nucleated osteoclasts were counted (**B**). Data are expressed as the percentages of the value of cells treated with RANKL (means ± standard deviations (SD), *n* = 3); TRAP activity was measured at λ = 405 nm (**C**). Data are expressed as percentages of the values of untreated cells (means ± standard deviations, *n* = 3). Different corresponding letters indicate significant differences at *p* < 0.05 by Duncan’s test. ### *p* < 0.001 *vs.* C (C: control, which was not treated; TC: treated control, which was treated with RANKL).

Osteoclast cells are responsible for bone resorption in the bone remodeling process and are coupled tightly with osteoblasts in order to form a new bone matrix. Excessive bone resorption by osteoclasts can result in bone loss, causing diseases, such as osteoporosis and rheumatoid arthritis [13]. In the present study, the inhibitory effects of genistein on osteoclast differentiation were investigated to establish their antiosteoporotic activity through suppressing excessive bone resorption by osteoclasts. Osteoclast differentiation from murine macrophage RAW 264.7 cells was induced by RANKL, which is essential for the terminal differentiation of monocytes/macrophages into osteoclasts [15]. Genistein at 1–20 μm was not toxic to RAW 264.7 macrophage cells during a period of five days of differentiation (data not shown). TRAP-positive multinucleated osteoclasts were visualized by light microphotography (Figure 2A). RANKL treatment induced osteoclast formation from RAW 264.7 pre-osteoclast cells and dramatically enhanced TRAP activity up to 297.9%, as shown in Figure 2A,C. Treatment with genistein reduced the number of multinucleated TRAP-positive cells and TRAP activity in a dose-dependent manner (Figure 2B,C). The inhibitory effects of natural flavonoids, such as luteolin [24], baicalein [25], resveratrol [26], epigallocatechin-3-gallate [27], scopoletin [28], curcumin [29] and fisetin [30], as potent antioxidants on osteoclast differentiation from osteoclastic precursors through controlling ROS generation, have also been reported. The suppressive effect on osteoclastogenesis via attenuating ROS production in RANKL-treated RAW 264.7 cells was observed in luteolin, which showed potent cellular antioxidant activity in rat C6 astroglioma cells [22,24]. Scopoletin at 1–10 µM also alleviated general ROS and superoxide anions produced in RANKL-treated RAW 264.7 cells to inhibit osteoclastic differentiation [28]. In addition, fisetin inhibited osteoclastogenesis through the prevention of RANKL-induced ROS production by nuclear transcription factor-erythroid 2-related factor 2 (Nrf2)-mediated upregulation of phase II antioxidant enzymes [30]. Thus, this study’s data support the hypothesis that the suppressive effect of genistein on RANKL-induced osteoclast differentiation may be closely related with its intracellular antioxidant activity.

### 2.3. The Suppressive Effect of ROS Level by Genistein during the Osteoclastic Differentiation of RAW 264.7 Cells

**Figure 3:**
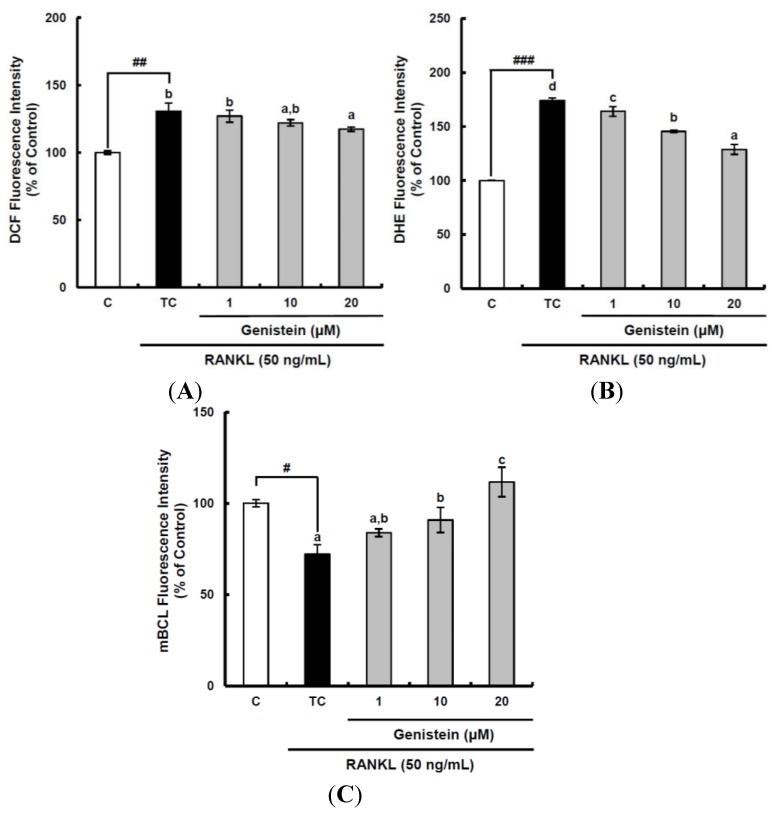
The suppressive effects of genistein on general ROS (**A**), superoxide anions (**B**) and GSH levels (**C**) during the receptor activator of nuclear factor kappa-B ligand (RANKL)-induced osteoclast differentiation. RAW 264.7 cells were seeded in 12-well plates (3 × 10^4^ cells/well) containing DMEM medium plus 10% FBS and incubated for 24 h. The medium was then replaced with a differentiation medium containing 50 ng/mL RANKL, and genistein at 1–10 µM was tested. After two days of incubation, the wells were gently washed twice with PBS. HBSS, which is fluorescently stable, was then added to each well instead of normal medium. DCFH-DA, dihydroethidium (DHE) or monochlorobimane (mBCl) was added to the culture plates at a final concentration of 40, 50 or 50 μM, respectively, and incubated for 30 min at 37 °C in the dark. After the cells were washed twice with HBSS, the fluorescence intensity of 2',7'-dichlorofluorescein (DCF), DHE and mBCl were measured using a Tecan GENios fluorometric plate reader. Data are expressed as the percentages of the value of untreated cells (means ± standard deviations, *n* = 3). Different corresponding letters indicate significant differences. Different corresponding letters indicate significant differences at *p* < 0.05 by Duncan’s test. # *p* < 0.05, ## *p* < 0.01, ### *p* < 0.001 *vs.* C (C: control, which was not treated; TC: treated control, which was treated with RANKL).

Accumulating evidence indicates that ROS are not only dangerous byproducts of cellular metabolism, but also important factors of signaling pathways in various cellular functions, including regulation, apoptosis and differentiation [31]. Previous studies have reported that the intracellular ROS level increases during RANKL-induced osteoclast differentiation, and this ROS generation can be attenuated by several antioxidants, including curcumin and fisetin [29,30]. Our previous study also showed that the intracellular ROS level in RANKL-stimulated Raw 264.7 cells increased to its largest value within two days and then declined [28]. Thus, to assess the suppressive effects of genistein on the production of different ROS during two days of incubation, the cell-permeable, fluorescent probes, DCFH-DA and dihydroethidium (DHE), were used. After DCFH-DA is permeated into the cell membrane, it is going to be hydrolyzed by esterase to release DCFH. DCFH then is easily oxidized by different kinds of ROS, including the peroxyl radical, hydrogen peroxide and peroxynitrite, except superoxide anions, to become fluorescent DCF. Accordingly, the fluorescence due to DCF formation can provide information on the intracellular oxidative stress level caused by different ROS, but cannot differentiate the species of ROS. On the other hand, DHE specifically reacting with superoxide anions can demonstrate the amount of superoxide anions intracellularly generated. The general ROS level determined by DCFH-DA was significantly (*p* < 0.01) increased by RANKL treatment to 130.6%, as compared to that of the control, and declined by genistein at 20 μm to 117.3% (Figure 3A). The superoxide anion level was also significantly (*p* < 0.001) augmented by 174.1% when compared to that in the control following RANKL stimulation and suppressed by genistein at 1–10 µM to 128.9% in a dose-dependent manner (Figure 3B). It is known that glutathione (GSH) exists in a high concentration (mm) depending upon the types of cell and tissue in comparison with other antioxidants and is involved in the front line of cellular antioxidant defense system. Therefore, GSH as a redox status biomarker has a vital role in the antioxidant defense system in cells. The GSH level was analyzed using monochlorobimane (mBCL), a fluorescent probe that conjugates with GSH. As expected, RANKL treatment significantly (*p* < 0.05) induced depletion in GSH level to 72.1%, as compared to that of the control (Figure 3C). However, genistein at 1–20 µM significantly (*p* < 0.05) enhanced the cellular GSH level depleted by RANKL treatment in a dose-dependent manner. These results imply that the suppressive effect of genistein on general ROS and superoxide anions level is likely to be potent, which is similar to its inhibitory effects on the RANKL-induced differentiation of RAW 264.7 cells into osteoclasts, indicating that genistein may suppress osteoclast differentiation from osteoclastic precursors through controlling ROS level.

### 2.4. The Inhibitory Effect of Genistein on the Translation and Activation of Nox1 and the Disruption of the Mitochondrial Electron Transport Chain System

**Figure 4:**
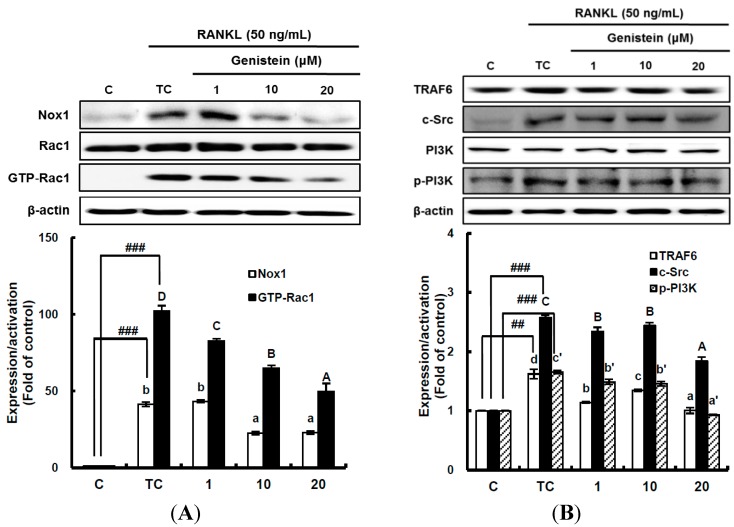
The effect of genistein on the translation and activation of NADPH oxidase (Nox1) (**A**) and its upstream signals (**B**). RAW 264.7 cells were exposed to the receptor activator of nuclear factor kappa-B ligand (RANKL) (50 ng/mL) for two days in the absence and presence of genistein. GTP-Rac1 was recovered by centrifugation from proteins complexed to the beads, which have a glutathione *S*-transferase-p21-binding domain: human Pak1 as 67–150 and binds to GTP-bound Rac1 of the lysates. Western blotting analysis was performed on the lysates of cells that had been incubated with 1–20 μm genistein. Different corresponding letters indicate significant differences at *p* < 0.05 by Duncan’s test. ## *p* < 0.01, ### *p* < 0.001 *vs.* C (C: control, which was not treated; TC: treated control, which was treated with RANKL).

It has been suggested that ROS-mediated RANKL-induced differentiation of RAW 264.7 cells into osteoclasts occurs through the activation of Nox homologues and the electron transport chain in mitochondria [4,8]. This means that superoxide anions are first produced by Nox and the mitochondrial electron transport chain system as ROS during osteoclast differentiation. Thus, to determine whether genistein suppresses superoxide anion production by Nox1, the translation and activation level of Nox1 was analyzed by western blotting. The translation level of Nox1, known as a major contributing factor in superoxide anion generation, was significantly (*p* < 0.01) stimulated by 50 ng/mL RANKL treatment, but it was significantly (*p* < 0.05) attenuated by genistein at 1–10 μm in a dose-dependent manner (Figure 4A). The small guanosine triphosphatase (GTPase) Rac1 existing as a cytosolic component of Nox1 in macrophages is essential for the activation of Nox1. Therefore, to examine the effect of genistein on Nox1 activation, the guanosine triphosphate (GTP) bound Rac1 (GTP-Rac1) and Rac1 levels were determined [6]. As seen in Figure 4A, Rac1 was not changed, but GTP-Rac1 significantly (*p* < 0.001) augmented by the RANKL treatment was significantly (*p* < 0.05) reduced by genistein at 1–20 μm in a dose-dependent manner, suggesting that genistein ameliorates Nox1 activation via controlling the GTP-Rac1 level. In addition, upstream signals, such as tumor necrosis factor receptor-associated factor (TRAF 6), tyrosine kinase c-Src, and phosphatidylinositol 3-kinase (PI3K), which are upregulated by RANKL and required for Nox1 activation, were analyzed. As shown in Figure 4B, TRAF6 expression significantly (*p* < 0.01) induced by RANKL treatment was ameliorated by genistein at 10–20 μm. c-Src expression significantly (*p* < 0.001) augmented by RANKL treatment was also markedly attenuated by genistein at 10–20 μm in a dose-dependent manner when compared to the treated control, and PI3k activation significantly (*p* < 0.001) induced by RANKL treatment was dose-dependently downregulated by genistein at 10–20 μm. These data indicate that genistein controls ROS generation by attenuating Nox1 translation, as well as Nox1 activation via the TRAF6/cSrc/PI3k signaling pathway in RANKL-mediated osteoclast differentiation from RAW 264.7 cells.

**Figure 5:**
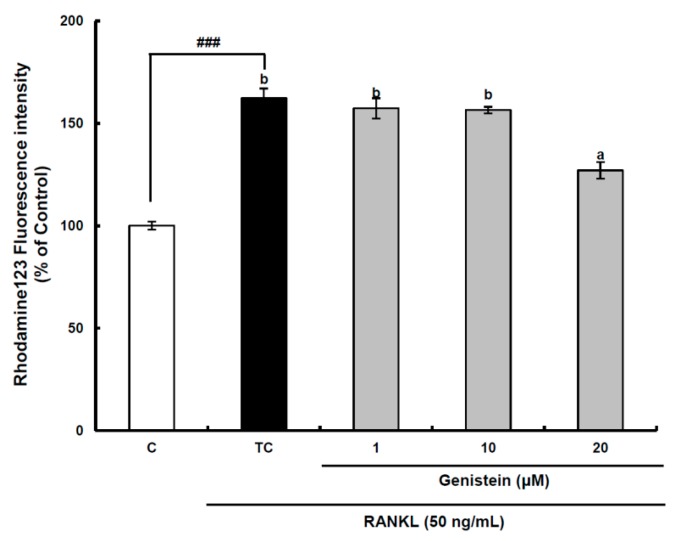
The effect of genistein on mitochondrial membrane potential in RANKL-treated Raw264.7 cells. RAW 264.7 cells were exposed to the receptor activator of nuclear factor kappa-B ligand (RANKL) (50 ng/mL) for two days in the absence and the presence of genistein. After two days of incubation, the wells were gently washed twice with PBS. HBSS, which is fluorescently stable, was then added to each well. Rhodamine 123, a fluorescent probe detecting mitochondrial membrane potential, was added to the culture plates at a final concentration of 5 µM and incubated for 30 min at 37 °C in the dark. After the cells were washed with HBSS, the fluorescence intensity of rhodamine 123 was measured with an excitation wavelength of 485 nm and an emission wavelength of 535 nm using a Tecan GENios fluorometric plate reader. Data are expressed as percentages of the values of untreated cells (means ± standard deviations, *n* = 3). Different corresponding letters indicate significant differences at *p* < 0.05 by Duncan’s test. ### *p* < 0.001 *vs.* C (C: control, which was not treated; TC: treated control, which was treated with RANKL).

Superoxide anions are also produced as a normal metabolic byproduct of the electron transport chain in mitochondria. Previous studies have shown that intra-mitochondrial ROS generation can disrupt the mitochondrial membrane potential to induce a retrograde signaling pathway resulting in osteoclastogenesis in addition to mitochondrial dysfunction [8,32]. Therefore, the mitochondrial membrane potential was measured using a stable fluorescence probe, rhodamine 123 [33]. In the assay system, the fluorescence value measured is inversely proportional to the mitochondrial membrane potential, because most of the rhodamine 123 added to RAW 264.7 cells without RANKL treatment is concentrated in the mitochondrial matrix and quenched, resulting in a low fluorescence value. The mitochondrial membrane potential of preosteoclastic precursor Raw264.7 cells was significantly (*p* < 0.001) decreased by RANKL treatment, but it was significantly (*p* < 0.05) recovered by the 20-μm genistein treatment (Figure 5), indicating that mitochondrial ROS generation contributing to osteoclast differentiation may be attenuated by genistein. However, a further study is required for a clear understanding of the inhibitory effect of genistein on the disruption of the mitochondrial electron transport chain system in the osteoclast differentiation of RAW 264.7 cells by RANKL treatment. Overall, in view of controlling ROS generation in RANKL-treated RAW 264.7 cells, the above results provide solid evidence for the modulation of genistein on the superoxide anion level through suppressing both the translation and activation of Nox1 and protecting the disruption of the mitochondrial electron transport chain system.

**Figure 6:**
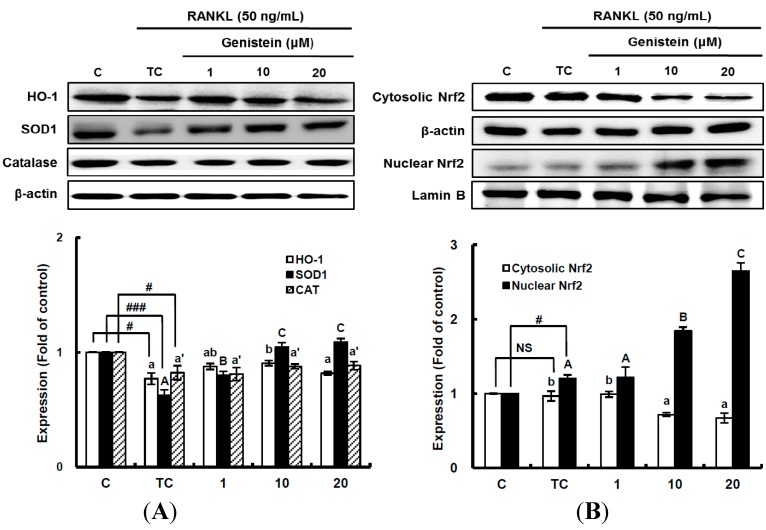
The effects of genistein on the expression of detoxifying and antioxidant enzymes (**A**) and the activation of Nrf2 (**B**) in RANKL-treated RAW 264.7 cells. RAW 264.7 cells were exposed to the receptor activator of nuclear factor kappa-B ligand (RANKL) (50 ng/mL) for two days in the absence and presence of genistein. RAW 264.7 cells were lysed in radioimmunoprecipitation assay (RIPA) buffer (50 mM Tris-HCl (pH 8.0), 1% nonyl phenoxypolyethoxylethanol (NP)-40, 0.5% sodium deoxycholate, 150 mM NaCl and 1 mM phenylmethylsulfonyl fluoride (PMSF)) that contained a phosphatase inhibitor cocktail. The nuclear proteins in the RAW 264.7 cells were extracted by using Buffer A (10 mM hydroxyethyl piperazineethanesulfonic acid (HEPES), pH 7.9), 10 mM KCl, 2 mM MgCl_2_, 1 mM dithiothreitol (DTT), 0.5 mM ethylenediamine tetra-acetic acid (EDTA) (pH 7.9), 0.1 μM PMSF and 1× protease inhibitor cocktail) and Buffer B (50 mM HEPES (pH 7.9), 50 mM KCl, 0.3 mM NaCl, 1 mm DTT, 1 mM EDTA (pH 7.9), 0.1 μM PMSF and 10% glycerol). Western blotting analysis was performed on the lysates of cells that had been incubated with 1–20 μm genistein. Different corresponding letters indicate significant differences at *p* < 0.05 by Duncan’s test. # *p* < 0.05, ### *p* < 0.001 *vs.* C (C: control, which was not treated; TC: treated control, which was treated with RANKL; NS: not significant).

### 2.5. The Enhanced Nrf2-Mediated Translation of Phase II Antioxidant Enzymes by Genistein

After superoxide anions are produced, they can be transformed into different ROS, such as hydrogen peroxide and peroxynitrite. Previous studies suggest that after the superoxide anions generated by RANKL treatment in RAW 264.7 cells may be transformed by SOD into hydrogen peroxide, which may be immediately catalyzed by catalase, resulting in water and oxygen [5,28]. Next, to understand the suppressive effects of genistein on the ROS and superoxide anion level by inducing antioxidant enzymes, the translation level of SOD1 and catalase were analyzed by western blotting. As shown in Figure 6A, the 50-ng/mL RANKL treatment significantly (*p* < 0.001) suppressed SOD1 expression, but genistein at 1–10 μm dose-dependently rescued SOD1 expression as compared to the treated control. In addition, regarding another important antioxidant enzyme, HO-1 expression significantly (*p* < 0.05) alleviated by RANKL treatment was recovered by genistein at 1–10 μm in a dose-dependent manner (Figure 6A). However, catalase expression significantly (*p* < 0.05) reduced by RANKL treatment was not significantly (*p* < 0.05) changed by genistein at 1–20 μm when compared to the treated control. These results indicate that genistein enhances SOD1 and HO-1 expression to result in the suppression of ROS and superoxide anion production in RANKL-treated RAW 264.7 cells. This upregulating effect on HO-1 expression in RANKL-treated RAW 264.7 cells was also observed in fisetin [22]. In the next experiment, the effect of genistein treatment on the activation of Nrf2 as an inducer of phase II antioxidant enzymes, including SOD1 and HO-1, was then analyzed (Figure 6B). As expected, the nuclear Nrf2 level was significantly (*p* < 0.05) elevated by genistein at 1–20 μm in a dose-dependent manner, proving that Nrf2 was translocated to a nucleus after being activated by genistein. This result is in good agreement with previous studies showing the Nrf2-driven induction of antioxidant enzyme genes by phytochemicals [34,35,36]. Accordingly, in RANKL-treated RAW 264.7 cells, genistein induces the activation of Nrf2, one of the ROS-sensitive nuclear factors leading to the translocation to a nucleus, and contributes to the enhanced expression of phase II antioxidant enzyme genes, such as SOD1 and HO-1.

## 3. Experimental Section

### 3.1. Materials and Reagents

Genistein, 2,2'-azobis(2-amidinopropane) dihydrochloride (AAPH), Trolox, quercetin, neocuproine, 3-(4,5-dimethylthiazol-2-yl)-2,5-diphenyltetrazolium bromide (MTT), 1,10-phenanthroline, Dulbecco’s modified Eagle’s medium (DMEM), Hank’s balanced salt solution (HBSS), phosphate-buffered saline (PBS, pH 7.4), a leukocyte acid phosphatase assay kit, sodium tartrate, *p*-nitro phenylphosphate (PNPP), RANKL, 2',7'-dichlorodihydrofluorescein-diacetate (DCFH-DA), dihydroethidium (DHE), monochlorobimane (mBCl), rhodamine 123, dimethylsulfoxide (DMSO) and fetal bovine serum (FBS) were purchased from Sigma Co. (St. Louis, MO, USA). Antibodies purchased from Santa Cruz Biotechnology (Santa Cruz, CA, USA) included Rac1, TRAF6, c-Src, PI3K, HO-1, SOD1, Nrf2 and catalase. Anti-Nox1 was purchased from Abcam (Cambridge, UK). Anti-SOD1 and p-PI3K were purchased from Cell Signaling Technology (Beverly, MA, USA). Human hepatoma (HepG2) cells and RAW 264.7 cells were obtained from the American Type Culture Collection (ATCC, Rockville, MD, USA).

### 3.2. Cellular Antioxidant Capacity

Cellular oxidative stress due to ROS generated by AAPH or H_2_O_2_ was measured spectrofluorometrically using the DCFH-DA method [37]. DCFH-DA diffuses through the cell membrane and is enzymatically hydrolyzed by intracellular esterase to non-fluorescent 2',7'-dichlorofluorescein (DCFH), which is rapidly oxidized to highly fluorescent DCF in the presence of ROS. HepG2 cells were first cultured in 96-well plates (5 × 10^5^/mL) with DMEM for 24 h. After the cells were incubated with different concentrations of sample dissolved in DMSO for 30 min, the medium was discarded, and the wells were gently washed twice with PBS. HBSS, which is fluorescently stable, was then added to each well instead of normal medium, and AAPH or H_2_O_2_ was used as an oxidative stress inducer. After the cells were treated with 60 μM AAPH or 1 mM H_2_O_2_ for 30 min, DCFH-DA was added to the culture plates at a final concentration of 40 µM and the cells incubated for 30 min at 37 °C in the dark. After incubation, the cells were washed with HBSS, and DCF fluorescence intensity was measured at an excitation wavelength of 485 nm and an emission wavelength of 535 nm using a Tecan (Salzburg, Austria) GENios fluorometric plate reader.

### 3.3. Cell Culture

Murine macrophage RAW 264.7 (ATCC, TIB-71) cells were purchased from the ATCC (Rockville, MD, USA) and cultured in DMEM supplemented with 10% (*v*/*v*) FBS and 1% (*v*/*v*) antibiotics in a humidified atmosphere of 5% CO_2_ at 37 °C.

### 3.4. Cell Cytotoxicity by MTT Assay

RAW 264.7 cells were cultured in 24-well plates (2 × 10^4^ cells/mL) for 5 days, washed with PBS and pretreated with different concentrations (1–20 μM) of genistein to be tested. After 5 days of incubation, MTT reagent was added to each well, and the plates were incubated at 37 °C for 1 h. The medium was removed, and the plates were washed twice with PBS. The intracellular insoluble formazan was dissolved in DMSO. The absorbance of each cell was recorded in DMSO. The absorbance of each cell was then measured at 570 nm using an ELISA (Tecan, Salzburg, Austria) reader, and the percentage of proliferation was calculated.

### 3.5. TRAP Staining

RAW 264.7 cells were seeded in 12-well plates (3 × 10^4^ cells/well) containing DMEM medium plus 10% FBS, and the medium was replaced with test samples in differentiation medium containing 50 ng/mL RANKL. The differentiation medium was changed every 2 days. After 5 days, the medium was removed, and the cell monolayer was gently washed twice using PBS. The cells were fixed in 3.5% formaldehyde for 10 min and washed with distilled water. The cells were incubated at 37 °C in a humid and light-protected incubator for 1 h in the reaction mixture of a leukocyte acid phosphatase assay kit (Sigma-Aldrich, St. Louis, MO, USA, Cat. No. 387), as directed by the manufacturer. The cells were washed three times with distilled water, and TRAP-positive multinucleated cells containing three or more nuclei were counted under a light microscope.

### 3.6. TRAP Activity

After differentiating the RAW 264.7 cells into osteoclasts for 5 days, the medium was removed, and the cell monolayer was gently washed twice using ice-cold PBS. The cells were fixed in 3.5% formaldehyde for 10 min and ethanol/acetone (1:1) for 1 min. Subsequently, the dried cells were incubated in a 50-mM citrate buffer (pH 4.5) containing 10 mM sodium tartrate and 6 mM PNPP. After 1 h of incubation, the reaction mixtures were transferred to new well plates containing an equal volume of 0.1 N NaOH. Absorbance was measured at 405 nm using an enzyme-linked immunoassay reader, and TRAP activity was expressed as the percent of the untreated control.

### 3.7. Detection of Intracellular ROS and Superoxide Anions

Fluorescent probes, such as DCFH-DA (excitation (Ex)/emission (Em) = 485 nm/535 nm) and DHE (Ex/Em = 518 nm/605 nm), which react specifically with ROS, were used to investigate the scavenging capacity of genistein against general ROS and superoxide anions [38]. RAW 264.7 cells were seeded in 12-well plates (3 × 10^4^ cells/well) containing DMEM medium plus 10% FBS and incubated for 24 h. The medium was then replaced with a differentiation medium containing 50 ng/mL RANKL, and the samples were tested. After 2 days of incubation, the medium was discarded, and the wells were gently washed twice with PBS. HBSS, which is fluorescently stable, was then added to each well instead of normal medium. DCFH-DA or DHE was added to the culture plates at a final concentration of 40 and 50 μM, respectively, and incubated for 30 min at 37 °C in the dark. After the cells were washed twice with HBSS, the fluorescence intensity of DCF and DHE were measured using a Tecan GENios fluorometric plate reader.

### 3.8. Measurement of Intracellular GSH

RAW 264.7 cells were seeded in 12-well plates (3 × 10^4^ cells/well) containing DMEM medium plus 10% FBS and incubated for 24 h. The medium was then replaced with a differentiation medium containing 50 ng/mL RANKL, and the samples were tested. After 2 days of incubation, the medium was discarded, and the wells were gently washed twice with PBS. HBSS, which is fluorescently stable, was then added to each well instead of normal medium. mBCL, a fluorescent probe that conjugates with GSH, was added to the culture plates at a final concentration of 50 µM and incubated for 30 min at 37 °C in the dark [39]. After the cells were washed with HBSS, the fluorescence intensity of mBCL was measured with an excitation wavelength of 380 nm and an emission wavelength of 465 nm using a Tecan GENios fluorometric plate reader.

### 3.9. Measurement of Mitochondrial Membrane Potential

RAW 264.7 cells were seeded in 12-well plates (3 × 10^4^ cells/well) containing DMEM medium plus 10% FBS and incubated for 24 h. The medium was then replaced with a differentiation medium containing 50 ng/mL RANKL, and the samples were tested. After 2 days of incubation, the medium was discarded, and the wells were gently washed twice with PBS. HBSS, which is fluorescently stable, was then added to each well instead of normal medium. Rhodamine 123, a fluorescent probe detecting mitochondrial transmembrane permeability, was added to the culture plates at a final concentration of 5 µM and incubated for 30 min at 37 °C in the dark. After the cells were washed with HBSS, the fluorescence intensity of rhodamine 123 was measured with an excitation wavelength of 485 nm and an emission wavelength of 535 nm using a Tecan GENios fluorometric plate reader [33].

### 3.10. Western Blot Analysis

RAW 264.7 cells were seeded in 12-well plates (3 × 10^4^ cells/well) containing DMEM medium plus 10% FBS and incubated for 24 h. The medium was then replaced with a differentiation medium containing 50 ng/mL RANKL, and the samples were tested. After 2 days of incubation, media were discarded, and the wells were gently washed twice with PBS. RAW 264.7 cells were lysed in radioimmunoprecipitation assay (RIPA) buffer (50 mM Tris-HCl (pH 8.0), 1% NP-40, 0.5% sodium deoxycholate, 150 mm NaCl, and 1 mm PMSF) that contained a phosphatase inhibitor cocktail. The nuclear proteins in the RAW 264.7 cells were extracted by using Buffer A (10 mm HEPES (pH 7.9), 10 mm KCl, 2 mM MgCl_2_, 1 mm DTT, 0.5 mm EDTA (pH 7.9), 0.1 μm PMSF and 1× protease inhibitor cocktail) and Buffer B (50 mm HEPES (pH 7.9), 50 mm KCl, 0.3 mm NaCl, 1 mm DTT, 1 mm EDTA (pH 7.9), 0.1 μm PMSF and 10% glycerol). The lysed cells were then subjected to electrophoresis using sodium dodecyl sulfate-polyacrylamide gel electrophoresis (SDS-PAGE) and transferred to nitrocellulose membranes. The membranes were reacted with primary antibodies for 12 h and then incubated with the appropriate horseradish peroxide-conjugated secondary antibodies for 1 h at room temperature. The proteins on the membranes were detected with a chemiluminescent detection kit (Intron Biotechnology, Seongnam, Korea) and visualized using an LAS4000 chemiluminescent image analyzer (Fuji, Tokyo, Japan).

### 3.11. Statistical Analysis

All data are presented as means ± SD. Statistical analyses were carried out using the statistical package SPSS (Statistical Package for Social Science, SPSS Inc., Chicago, IL, USA) program, and the significance of each group was verified with one-way ANOVA followed by the Student’s *t-*test.

## 4. Conclusions

The intracellular ROS level can be maintained as a result of ROS generation and removal. In this study, it was confirmed that genistein suppresses osteoclast differentiation of RANKL-treated RAW 264.7 cells through attenuating the ROS level. Whether genistein can inhibit the translation and activation of Nox1 and the disruption of the mitochondrial electron transport chain system and whether genistein can induce the expression of phase II antioxidant enzymes, such as SOD1 and HO-1, were investigated. Overall, this study’s results provide a conclusive explanation that the inhibitory effects of genistein on RANKL-stimulated osteoclast differentiation is due to the control of ROS production through suppressing the translation and activation of Nox1 and the disruption of the mitochondrial electron transport chain system, as well as ROS scavenging through Nrf2-mediated induction of phase II antioxidant enzymes, such as SOD1 and HO-1.

Furthermore, soy-based products containing genistein may be used for the development of potential therapeutic agents preventing bone diseases, such as osteoporosis and rheumatoid arthritis, through attenuating osteoclast differentiation.
